# Development of a risk prediction model for radiation dermatitis following proton radiotherapy in head and neck cancer using ensemble machine learning

**DOI:** 10.1186/s13014-024-02470-1

**Published:** 2024-06-24

**Authors:** Tsair-Fwu Lee, Yen-Hsien Liu, Chu-Ho Chang, Chien-Liang Chiu, Chih-Hsueh Lin, Jen-Chung Shao, Yu-Cheng Yen, Guang-Zhi Lin, Jack Yang, Chin-Dar Tseng, Fu-Min Fang, Pei-Ju Chao, Shen-Hao Lee

**Affiliations:** 1 Medical Physics and Informatics Laboratory of Electronics Engineering, National Kaohsiung University of Science and Technology, No.415, Jiangong Rd., Sanmin Dist,, Kaohsiung, 807 Taiwan (ROC); 2 Graduate Institute of Clinical Medicine, Kaohsiung Medical University, Kaohsiung, 807 Taiwan (ROC); 3 Department of Medical Imaging and Radiological Sciences, Kaohsiung Medical University, Kaohsiung, 80708 Taiwan (ROC); 4 Medical Physics at Monmouth Medical Center, Barnabas Health Care, NJ Long Branch, US; 5 Department of Radiation Oncology, Kaohsiung Chang Gung Memorial Hospital, Chang Gung University College of Medicine, Kaohsiung, Taiwan (ROC); 6 Department of Radiation Oncology, Linkou Chang Gung Memorial Hospital, Chang Gung University College of Medicine, Linkou, Taiwan (ROC)

**Keywords:** Radiation dermatitis, Proton radiotherapy, Head and neck cancer, Ensemble machine learning, Predictive modeling, Feature selection

## Abstract

**Purpose:**

This study aims to develop an ensemble machine learning-based (EML-based) risk prediction model for radiation dermatitis (RD) in patients with head and neck cancer undergoing proton radiotherapy, with the goal of achieving superior predictive performance compared to traditional models.

**Materials and methods:**

Data from 57 head and neck cancer patients treated with intensity-modulated proton therapy at Kaohsiung Chang Gung Memorial Hospital were analyzed. The study incorporated 11 clinical and 9 dosimetric parameters. Pearson’s correlation was used to eliminate highly correlated variables, followed by feature selection via LASSO to focus on potential RD predictors. Model training involved traditional logistic regression (LR) and advanced ensemble methods such as Random Forest and XGBoost, which were optimized through hyperparameter tuning.

**Results:**

Feature selection identified six key predictors, including smoking history and specific dosimetric parameters. Ensemble machine learning models, particularly XGBoost, demonstrated superior performance, achieving the highest AUC of 0.890. Feature importance was assessed using SHAP (SHapley Additive exPlanations) values, which underscored the relevance of various clinical and dosimetric factors in predicting RD.

**Conclusion:**

The study confirms that EML methods, especially XGBoost with its boosting algorithm, provide superior predictive accuracy, enhanced feature selection, and improved data handling compared to traditional LR. While LR offers greater interpretability, the precision and broader applicability of EML make it more suitable for complex medical prediction tasks, such as predicting radiation dermatitis. Given these advantages, EML is highly recommended for further research and application in clinical settings.

## Introduction

Radiation therapy is a primary treatment for head and neck cancers, but it frequently leads to radiation dermatitis (RD), a side effect that ranges from mild erythema to severe ulceration, significantly affecting patients’ quality of life and treatment outcomes [1]. Given its high prevalence and impact, RD is the focal endpoint of this study. Understanding and predicting this common and disruptive side effect is crucial for enhancing patient management and refining treatment protocols.

Analyzing prognostic factors for RD through data analytics and modeling is essential [2, 3], particularly in the context of proton radiotherapy. Traditional Lyman-Kutcher-Burman (LKB) models, which assess dose-related complication risks, are limited as they consider only single-dose thresholds and neglect potential clinical factors [4]. Conversely, machine learning (ML) provides a robust, rapid, and flexible alternative for individualized risk assessment by integrating a broader spectrum of factors affecting side effects.

In previous work [5], we used Logistic Regression (LR) and Least Absolute Shrinkage and Selection Operator (LASSO) techniques to develop a predictive model for xerostomia incidence in intensity-modulated proton therapy, incorporating both dosimetric and clinical factors. However, the simplicity of LR limits its effectiveness in complex feature spaces [6], necessitating external methods like LASSO for dimensionality reduction. Additionally, LR’s linear nature restricts its ability to capture non-linear interactions. Thus, ML stands out as a promising upgrade over the traditional multivariate LR-based Normal Tissue Complication Probability (NTCP) and LKB models.

Unlike the LKB algorithm, which primarily focuses on dosimetric parameters, ML facilitates a more comprehensive analysis of features relevant to radiation therapy effects, leading to more precise predictions [4]. Through detailed data analysis, ML identifies various factors that could lead to side effects, covering both dosimetric and clinical variables. The ability to incorporate new datasets into the existing models not only enhances their robustness but also facilitates more personalized risk assessments.

Moreover, a newer approach known as Ensemble Machine Learning (EML) has gained prominence within ML. This advanced technique merges multiple base models, or weak learners, to create a stronger and more robust ensemble learner. Employing various ensemble methods like bagging, boosting, and stacking [7, 8] helps refine the system, with base models ranging from decision trees to support vector machines and logistic regression.

Considering these advancements, this study aims to develop an EML-based risk prediction model for RD following proton therapy in head and neck cancer patients, targeting superior predictive performance.

## Materials and methods

**Fig. 1:**
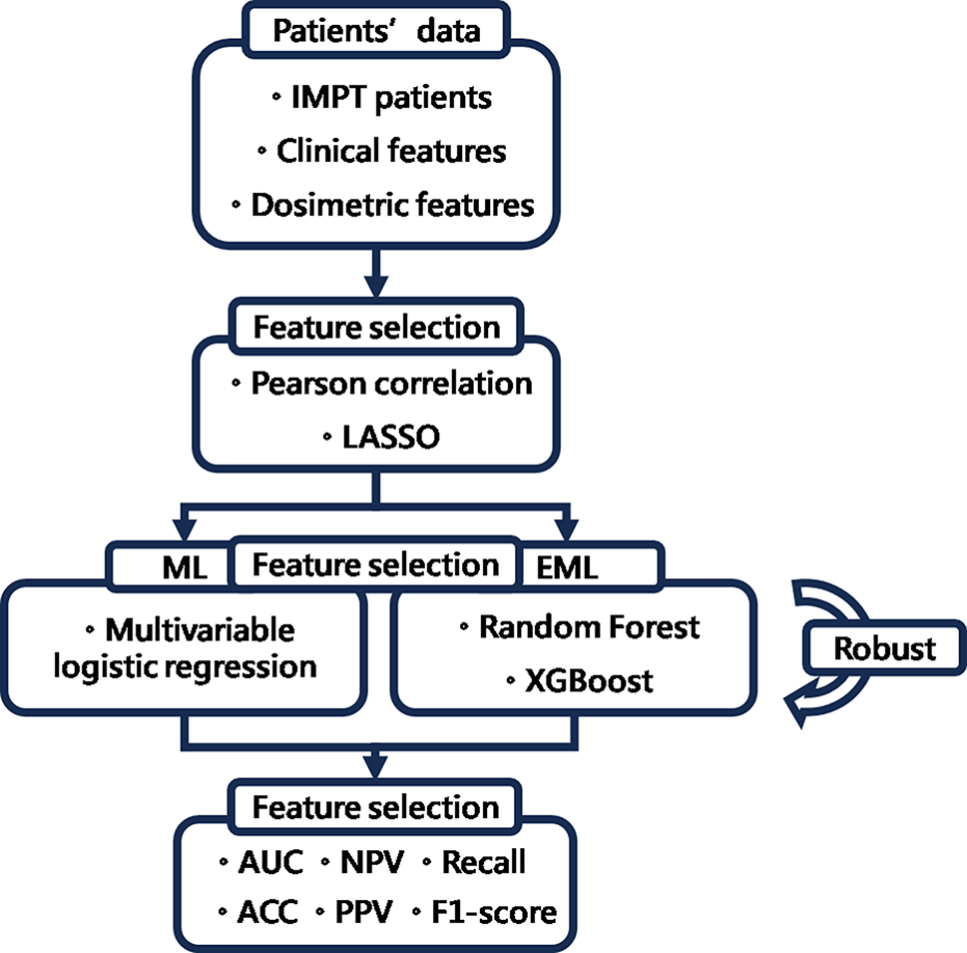
Research Workflow Diagram *Abbreviation* ML: machine learning; EML: ensemble machine learning; LASSO: least absolute shrinkage and selection operator; ACC: Accuracy; NPV: Negative predictive value; AUC: Area Under the Curve

This study employs data from 57 head and neck cancer patients, featuring 11 clinical factors and nine dosimetric parameters in treatment plans [9]. Considering two skin depths (3 and 5 mm) for complication assessment, a total of 18 dosimetric factors are analyzed. To mitigate collinearity and enhance model interpretability, Pearson’s correlation is used to eliminate highly correlated factors. LASSO is then applied for feature selection, focusing on variables potentially linked to radiation dermatitis. The models trained include both machine learning and ensemble learning techniques. Optimization is achieved through hyperparameter tuning, with performance metrics serving as the evaluation standard. Our research process, EML-based risk prediction model for RD, is depicted in Fig. 1.

In this study, data from NPC patients collected at Kaohsiung Chang Gung Memorial Hospital were used to develop and validate ML and EML models. Initially, feature selection was performed to identify variables most predictive of radiation dermatitis. The training and analysis were conducted using machine learning models that were implemented using Python and its libraries such as Scikit-learn for logistic regression and Random Forest, and XGBoost and CatBoost for ensemble methods. The models were trained within two different machine learning frameworks: traditional ML models and more advanced EML models, which use a multi-model ensemble technique to enhance prediction robustness and accuracy. Additionally, the EML models underwent robust optimization to ensure resilience to minor variations in input data, thereby enhancing reliability in practical applications. Finally, the effectiveness of these models was assessed through a series of performance evaluations, such as accuracy and AUC metrics. Hyperparameters were optimized using grid search techniques to identify the most effective settings for each model. This process not only improved the adaptability of the models to clinical practices but also enhanced the interpretability and accuracy of the predictions.

The analyses in this study were conducted using Python version 3.9.7 and SPSS software (Statistical Product and Service Solutions), version 25, developed by IBM (International Business Machines Corporation). The model fitting was conducted using Jupyter as the compilation software for Python 3.9.7. Machine learning models were implemented using their respective module packages; Random Forest and Logistic Regression models utilized the Scikit-learn package, while XGBoost and CatBoost were implemented using their official packages.

### Patients’ data

This research was authorized by the Institutional Review Board (IRB-201900736B0(201900736B0C102)). At the Kaohsiung Chang Gung Memorial Hospital in Taiwan, treatment for nasopharyngeal carcinoma (NPC) patients using intensity-modulated proton therapy (IMPT) commenced in January 2019 [9]. This study focused on patients newly diagnosed with NPC who underwent the complete IMPT treatment [9]. Exclusion criteria included patients who did not finish the treatment, those who experienced interruptions during the treatment, or had previously received radiotherapy in the head and neck area. Following approval by the IRB, 57 patients were selected for data analysis. Clinical and dosimetric factors potentially impacting radiation dermatitis were detailed in Tables 1 and 2. The median age of the patients at diagnosis was 49 (ranging from 31 to 71 years). The cohort consisted of 42 males and 19 smokers. According to the American Joint Committee on Cancer (AJCC) 8th edition, the staging distribution was 4 in stage I, 16 in stage II, 22 in stage III, and 15 in stage IVA. A significant majority, 91.2%, of the patients received IMPT in conjunction with chemotherapy [9]. Clinical factors assessed included cancer stage, lifestyle factors, and individual patient conditions, while dosimetric variables considered encompassed volumes and dose parameters at skin depths of 3 and 5 millimeters.

**Table 1:** Clinical features of patients

	< Grade 2 RD Value-x (%)	≥ Grade 2 RD Value-x (%)	*p*-value
	*n* = 26	*n* = 31	
AJCC Stage			0.007
I	4 (15)	0 (0)	
II	10 (38)	6 (19)	
III	5 (19)	17 (55)	
IV	7 (27)	8 (26)	
Tumor classification			0.661
T1	16 (62)	15 (48)	
T2	3 (12)	6 (19)	
T3	2 (8)	7 (23)	
T4	5 (19)	3 (10)	
Node classification			0.005
N0	6 (23)	3 (10)	
N1	13 (50)	8 (26)	
N2	6 (23)	12 (38)	
N3	1 (4)	8 (26)	
Age (years)			0.501
Mean	49	51	
Range	31–71	36–71	
< 49	14 (54)	16 (52)	
≥ 49	12 (46)	15 (48)	
BMI (kg/m^2^)			0.180
Mean	24.3	25.6	
Range	17.1–30.8	17.7–28.7	
< 24	13 (50)	10 (32)	
≥ 24	13 (50)	21 (68)	
Gender			0.193
Male	17 (65)	25 (81)	
Female	9 (35)	6 (19)	
DM			0.792
Yes	2 (8)	3 (10)	
No	24 (92)	28 (90)	
HTN			0.513
Yes	4 (15)	3 (10)	
No	22 (85)	28 (90)	
Smoking			0.008
Yes	4 (15)	15 (48)	
No	22 (85)	16 (52)	
Chemotherapy			0.499
Yes	23 (89)	29 (94)	
No	3 (11)	2 (6)	
Planning			0.188
Yes	15 (58)	23 (74)	
No	11 (42)	9 (26)	

*Abbreviations* AJCC: American Joint Committee on Cancer; RD: Radiation dermatitis; BMI: Body mass index; DM: Diabetes mellitus; HTN: Hypertension

**Table 2:** Dosimetric features of patients

Variable	< Grade 2 RD *n* = 26			≥ Grade 2 RD *n* = 31			*p*-value
	Mean	Range		Mean	Range		
Skin 3 mm							
V_40_ (ml)	58.26	23.26–116.06		65.62	23.44–155.41		0.040
V_50_	21.41	0.93–77.16		26.48	3.81–65.99		0.038
V_60_	1.49	0–9.12		4.79	0–30.17		0.021
V_70_	0.03	0–0.53		0.67	0–10.26		0.294
D_10_ (Gy)	52.06	44.53–59.85		54.78	45.84–70.11		0.025
D_30_	46.97	38.17–57.00		48.34	37.96–60.08		0.078
D_50_	42.00	33.24–54.19		42.95	32.85–52.41		0.068
D_70_	36.13	24.59–51.51		37.50	27.06–49.53		0.027
D_100_	28.44	17.76–44.06		30.20	21.48–46.44		0.020
Skin 5 mm							
V_40_ (ml)	104.61	52–199.16		116.08	46.47–237.43		0.035
V_50_	45.80	6.12–136.03		54.05	10.97–115.17		0.035
V_60_	4.37	0–25.49		10.55	0.15–47.61		0.008
V_70_	0.21	0–1.86		1.50	0–17.89		0.167
D_10_ (Gy)	55.74	48.60–64.13		58.69	50.38–71.76		0.014
D_30_	51.43	44.01–59.63		53.43	44.15–65.53		0.034
D_50_	48.53	40.33–58.03		49.93	39.30–59.28		0.062
D_70_	45.66	37.10–56.31		46.73	35.52–54.29		0.081
D_100_	40.49	29.69–53.75		41.87	31.85–51.12		0.037

*Abbreviations* RD: Radiation dermatitis; Vx: Volume receiving x Gy dose; Dx: Dose received by x cc volume

### Treatment

At Kaohsiung Chang Gung Memorial Hospital, nasopharyngeal carcinoma (NPC) is treated using intensity-modulated proton therapy (IMPT) with a Sumitomo Proton Machine, leveraging scanning beam technology. Treatment planning is performed on the RayStation treatment planning system (version 8, Raysearch Medical Laboratories, Stockholm, Sweden), typically utilizing three beam directions: left and right anterior obliques, and posterior fields, facilitated by multi-field optimization. To mitigate range and positional uncertainties, robust optimization techniques are employed, which include a 3.5% margin for range uncertainties and 3 mm for positional uncertainties. A comprehensive robust evaluation involves generating 21 scenario-based plans to determine the efficacy of each treatment setup. Treatment precision is further ensured through daily CT-based image guidance.

The treatment dosages are stratified by clinical target volume (CTV) classifications: the high-dose CTV (CTV-H) is prescribed 69.96 Gy, the medium-dose CTV (CTV-M) receives 59.4 Gy, and the low-dose CTV (CTV-L) varies between 52.8 and 54.0 Gy, delivered over 33 fractions at one fraction per day, five days a week. The CTV-H targets the primary tumor and associated lymph nodes with an isotropic expansion of 3 mm from the gross tumor volume (GTV). The CTV-M is designed to include adjacent at-risk anatomical structures such as the skull base and parapharyngeal space, addressing potential micro-metastatic pathways. The CTV-L covers subclinical lymphatic regions in the lower neck that are not directly tumor-involved [9].

Organs at risk (OARs) are meticulously outlined with specified dose constraints to minimize radiation exposure and mitigate side effects. These organs include the brainstem, brain, spinal cord, lens, optic nerve, optic chiasm, cochleas, thyroid gland, larynx, mandible, oral cavity, and the parotid and submandibular glands. The applied dose constraints adhere to established guidelines and recommendations, aiming to optimize treatment outcomes and reduce adverse effects [9].

### End points

In this study, we evaluated 57 follow-up assessments conducted during and 1 to 3 weeks after proton radiotherapy. The primary outcome was the occurrence of Grade 2 or higher radiation dermatitis (RD) as defined by the CTCAE 4.0 guidelines [10]. There were no reported cases of Grade 4 RD among the participants. The RD grading is as follows: Grade 1 consists of slight erythema or mild desquamation; Grade 2 involves more pronounced erythema, patchy moist desquamation mainly in skin folds, and moderate edema; Grade 3 includes widespread moist desquamation beyond skin folds, bleeding triggered by minor injuries; and Grade 4 is characterized by life-threatening effects, including dermis necrosis or ulceration, spontaneous bleeding at the affected site, and the potential need for skin grafting.

### Features selection

In feature selection, Pearson correlation coefficients were initially used to eliminate highly collinear variables, setting a threshold of 0.8 to define high correlation for exclusion. In cases of multicollinearity, the most statistically significant variable in univariate analysis (lowest p-value) was retained. After preliminary collinear feature removal, LASSO was employed to select remaining features. The feature set chosen by the LASSO model with the lowest prediction error was used as input parameters for subsequent predictive models.

### Ensemble models

To compare traditional machine learning with ensemble ML methods, two tree-based ensemble ML models were chosen based on feature types and non-linear modeling capabilities: random forest (RF) using bagging and XGBoost using boosting. Features were categorized into two sets: one using all remaining features after collinearity removal (RF_ALL_, XGB_ALL_), and the other using LASSO-selected features (RF_LASSO_, XGB_LASSO_), resulting in four models. For model interpretation, the SHAP method (SHapley Additive exPlanations) was employed to assess feature importance and contributions [11]. The best-performing model’s SHAP values were analyzed to evaluate each feature’s significance and impact on predicting radiation dermatitis.

### Robust optimization

For algorithm robust optimization, 10 iterations of 10-fold cross-validation [12] and grid search were employed to select the optimal hyperparameter combination for peak predictive performance, as depicted in the accompanying diagram. The dataset was initially split into an 80% training-validation set and a 20% test set. The grid search method exhaustively explored hyperparameter combinations across the training-validation set.

**Fig. 2:**
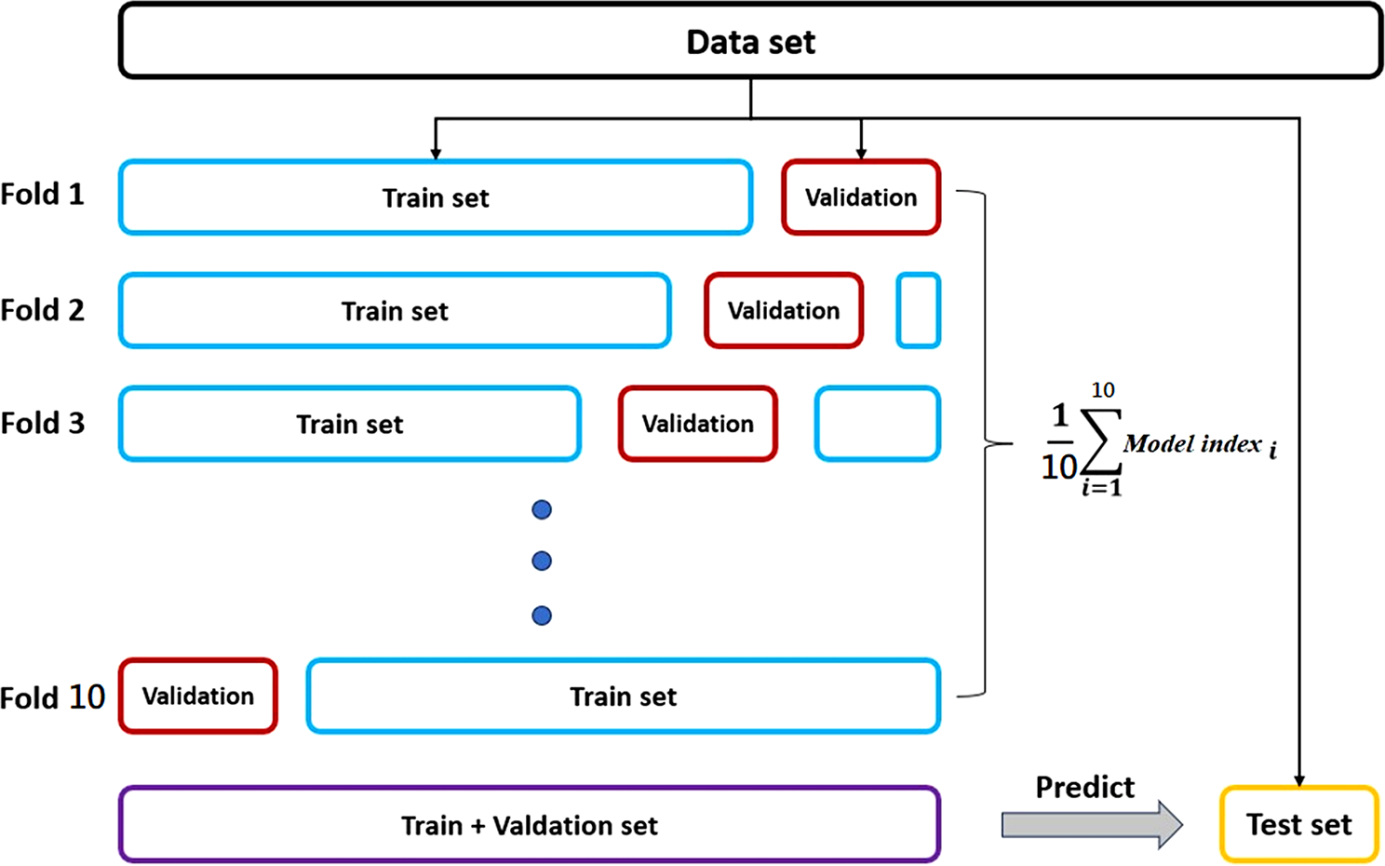
The structure of the cross-validation

The structure of the cross-validation is illustrated in the Fig. 2, where each fold in the 10-fold cross-validation process is used sequentially as a validation set while the remaining nine folds form the training set. This process is repeated across 10 iterations to mitigate the risk of overfitting and ensure robustness, especially in a smaller dataset. The performance from each validation fold is independently calculated and then averaged over all iterations to provide a reliable measure of model performance.

For the final model assessment, a unique single data point was randomly selected from the test set to act as an independent test instance. This focused approach provides a stringent test of the model’s predictive capability, assessing how well the model generalizes to new, unseen data. This rigorous testing methodology, which includes the systematic averaging of validation set performance and the final evaluation on a distinct test data point, ensures a comprehensive evaluation of the model’s generalization ability and predictive accuracy.

We acknowledge that a larger sample size would enhance the statistical power and generalizability of the results. However, this structured approach to validation and testing is designed to maximize the insights gained from the available data, thoroughly testing the model’s effectiveness across various subsets of the data and under different scenarios as outlined in the provided cross-validation diagram.

### Logistic regression model

Drawing on previous studies for constructing multivariate logistic NTCP models [5, 13, 14], this research establishes LR_LASSO_ as a comparative baseline against ensemble machine learning predictions. We employed the LASSO technique for automated feature selection, using the penalty term λ from the LASSO iteration with the lowest prediction error as the selection criterion. This approach identifies a feature set that balances predictive power and model simplicity for logistic regression modeling. Model performance is assessed based on area under the curve (AUC), R-squared, Omnibus test, and Hosmer-Lemeshow test.

## Results

**Fig. 3:**
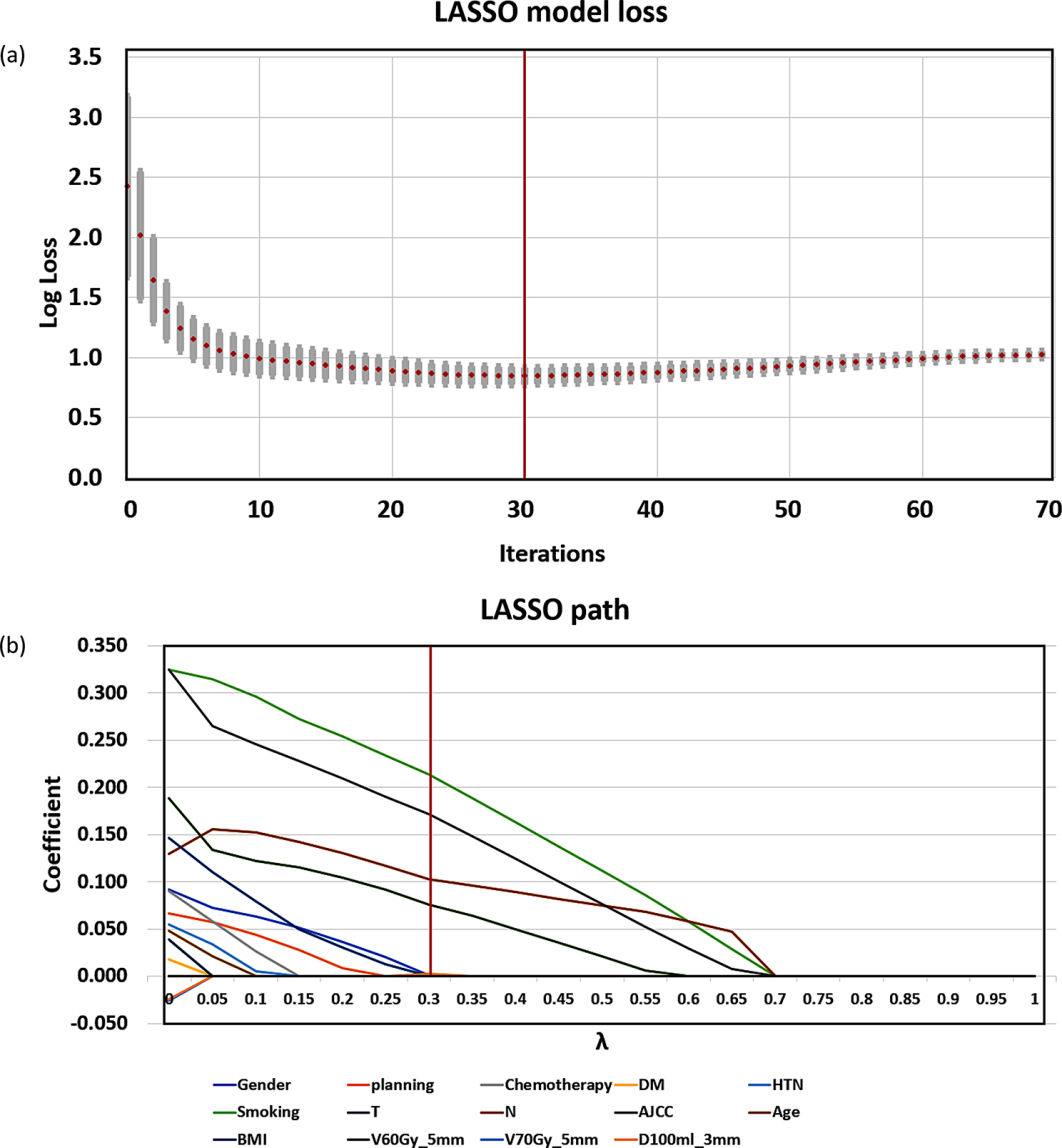
(**a**) Iterative Performance of LASSO Model with MAE Metrics (**b**) The LASSO shrinking path diagrams. *Abbreviation* LASSO: least absolute shrinkage and selection operator; ACC: Accuracy; NPV: Negative predictive value; AUC: Area Under the Curve; RD: Radiation dermatitis; BMI: Body mass index

Features with Pearson correlation coefficients above 0.8 were excluded, retaining only statistically significant variables with lower p-values in cases of multicollinearity. As a result, 11 clinical features were retained, while most dosimetric features were excluded, leaving only V60Gy_− 5 mm_, V70Gy_− 5 mm_, and D100_− 3 mm_. Prior to logistic regression, LASSO was used for feature selection. Figure 3(a) shows that the 30th LASSO iteration, with the lowest mean absolute error (MAE = 0.849), was used as the basis for feature selection for predicting radiation dermatitis. Post-LASSO, six features—Smoking, N stage, AJCC stage, V60_− 5 mm_, D100_− 3 mm_, and Gender—were retained based on their λ values, as shown in Fig. 3(b).

Table 3 presents the performance and coefficients of the logistic regression model built on six variables: Gender, Smoking, N stage, AJCC stage, V60_5mm, and D100_3mm. The table shows an AUC of 0.870, and other metrics like R^2^, Hosmer-Lemeshow test, and Omnibus test indicate a good model fit. Internal model parameters, such as coefficient β and odds ratio, reveal a significant correlation and causality between radiation dermatitis and smoking history. With a p-value < 0.05, the factor is statistically significant within the model. The positive coefficient β suggests a direct correlation between smoking history and radiation dermatitis, while the odds ratio indicates that smokers are 14.4 times more likely to develop radiation dermatitis than non-smokers.

**Table 3:** Performance Metrics and Parameters of LASSO-based Logistic Regression Model

AUC	ACC	*R* ^2^	Hosmer-Lemeshow	Omnibus	
0.870	73.7%	0.383	0.727	< 0.001	
Feature	$\beta$	p-value	OR (CI)	S.E.	Wald
Gender	1.427	0.178	4.166 (0.53–33.17)	1.06	1.82
Smoking	2.670	0.023	14.446 (1.47–143.30)	1.17	5.20
N	1.143	0.241	3.137 (0.46–21.23)	0.98	1.37
AJCC	2.335	0.065	10.325 (0.86–123.73)	1.27	3.39
V60_− 5 mm_	0.116	0.194	1.123 (0.94–1.34)	0.09	1.68
D100_− 3 mm_	-0.073	0.517	0.929 (0.745–1.160)	0.11	0.42
Constant	-2.223				

*Abbreviations* β: Coefficient; OR: Odds Ratio; CI: Confidence Interval; S.E.: Standard Error; AJCC: American Joint Committee on Cancer; N: Lymph Node Metastasis; Vx: Volume receiving x Gy dose; Dx: Dose received by x cc volume; ACC: Accuracy

In ensemble machine learning, two sets of features are used to build predictive models. The first set, post-collinearity exclusion, comprises 14 features and is used to construct RF_ALL_ and XGB_ALL_ models. The second set, selected by LASSO, includes six features: Gender, Smoking, N stage, AJCC stage, V60_− 5 mm_, and D100_− 3 mm_, and is used for RF_LASSO_ and XGB_LASSO_ models. The training-validation set primarily fine-tunes hyperparameters to prevent overfitting. Model performance is chiefly assessed by the test set’s AUC. Table 4 shows XGB_ALL_ outperforms XGB_LASSO_ with an AUC of 0.890 vs. 0.820, suggesting that more features may provide richer information or introduce noise that affects prediction. Conversely, RF_ALL_ and RF_LASSO_ show similar performance, indicating that feature selection has minimal impact on predictive accuracy.

**Table 4:** Evaluation Metrics for Two Predictive Models Built with and without LASSO Feature Selection Post-Collinearity Exclusion

Model	AUC	ACC	NPV	PPV	Recall	F1-Score
RF_ALL_	0.670	0.50	0.29	0.80	0.44	0.57
RF_LASSO_	0.710	0.50	0.29	0.80	0.44	0.57
XGB_ALL_	0.890	0.75	1.00	0.67	1.00	0.80
XGB_LASSO_	0.820	0.67	0.75	0.63	0.83	0.71

*Abbreviations* LR: Logistic regression; RF: Random forest; PPV: Positive predictive value; ACC: Accuracy; NPV: Negative predictive value

**Fig. 4:**
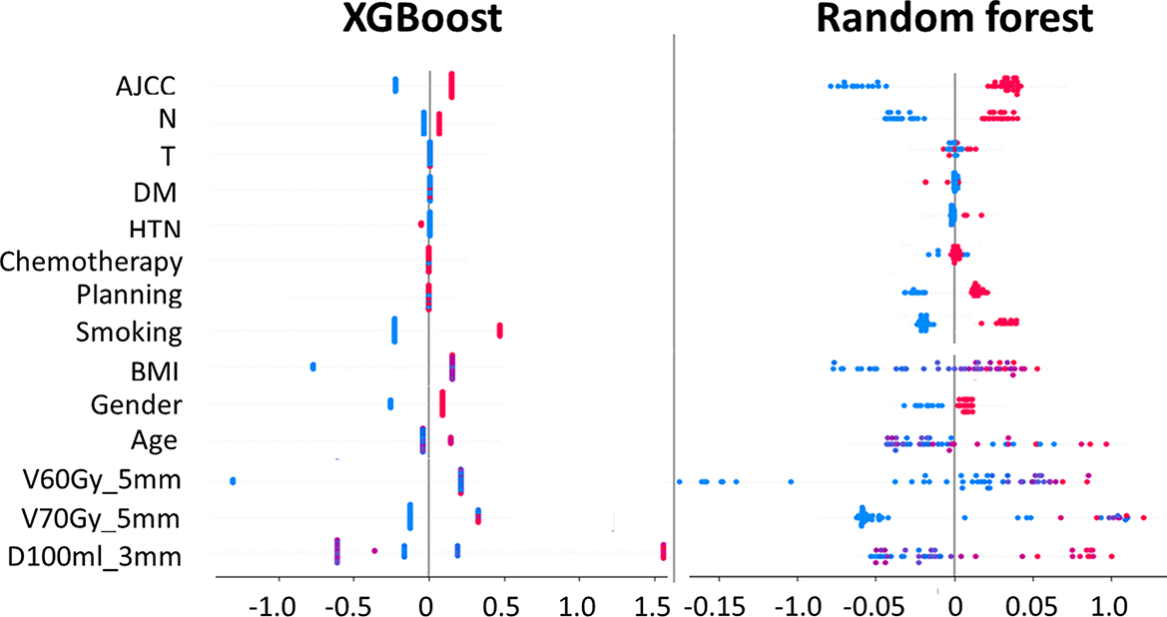
Bee Swarm Plot of Feature Importance via SHAP Values in Ensemble Learning Models. *Abbreviation* SHAP: SHapley Additive exPlanations; DM: Diabetes mellitus; HTN: Hypertension; RD: Radiation dermatitis; BMI: Body mass index

In terms of feature importance, SHAP values quantify each feature’s contribution to predictive performance after training. Higher SHAP values indicate a significant impact on predicting radiation dermatitis, while lower values suggest lesser relevance. Figure 4 reveals notable differences in feature importance between the XGBoost (XGB) and Random Forest (RF) models. For instance, the AJCC feature has a stronger impact in the XGB model, whereas in the RF model, its SHAP values are concentrated near zero. Similarly, the influence of smoking varies, with XGB displaying a moderate spread of values and RF showing less consistency.

The figure also uses color-coding for clarity—AJCC < 3 is shown in blue, ≥ 3 in red; non-smokers in blue, smokers in red. This color distribution forms two distinct clusters, clearly demonstrating the significant contribution of these features to the models’ ability to assess the severity of radiation dermatitis. Such distinct grouping highlights their predictive relevance and underscores their crucial role in determining the severity of skin reactions in patients.

## Discussion

This study employs Random Forest, XGBoost, and Logistic Regression to develop predictive models for assessing the risk of radiation dermatitis in head and neck cancer patients post-proton radiotherapy. Although Logistic Regression offers greater interpretability, machine learning models, particularly XGBoost, excel in predictive accuracy, aligning with the growing emphasis on machine learning for complication prediction in medical research. Studies like Poolakkad et al. [15], who achieved a higher AUC using gradient boosting for predicting mucositis post-chemotherapy, and Smith et al. [16], who demonstrated superior performance using neural networks for post-radiation xerostomia, support this trend. Dean et al.’s use of penalized logistic regression, SVM, and random forest to predict dysphagia with strong external validation further validates the efficacy of advanced models [16].

Additionally, Xie et al. [17] conducted a meta-analysis on risk factors for RD post-radiotherapy in breast cancer patients. Their sensitivity analysis revealed that European studies identified smoking as a risk factor for RD, while North American and Asian studies found no such correlation. Our study focuses on head and neck cancer patients, and according to Lilla et al. [18], the factors causing RD may vary based on the irradiation site. This could explain the discrepancy with Xie et al.‘s findings in breast cancer patients. Our data source, Fang et al. [19], his research also suggests a correlation between smoking and RD, aligning with our results. However, further validation is needed to confirm the link between smoking and RD, possibly due to data limitations.

In this study, logistic regression, a traditional machine learning method, is simpler but offers limited parameter tuning options, which can lead to overfitting and reduced generalization compared to ensemble methods. Ensemble machine learning methods aggregate predictions from multiple models, enhancing prediction accuracy, stability, and generalization capabilities [20, 21]. These methods are particularly effective in handling imbalanced datasets as they can improve minority class detection by balancing predictions from multiple models and are less likely to be biased towards the majority class. Furthermore, ensemble methods benefit from the diversity among weak learners, which can be fine-tuned through hyperparameter adjustments to optimize predictive performance [22]. Despite logistic regression showing comparable performance in some cases, the broader applicability and advanced capabilities of ensemble methods make them the preferred choice for complex predictive modeling tasks.

In our study, XGBoost significantly outperformed Random Forest, achieving a higher AUC score of 0.890 compared to Random Forest’s 0.670. This superior performance of XGBoost can be attributed to several key factors that enhance its robustness and adaptability. XGBoost integrates both L1 and L2 regularization techniques, which are crucial for preventing overfitting, especially beneficial in datasets with numerous features or smaller sample sizes [23]. Moreover, XGBoost utilizes the Gradient Boosting algorithm, which optimizes performance by building trees sequentially to correct errors from previous trees, allowing for more precise and adaptive parameter adjustments. This methodical approach to error correction and regularization significantly contributes to the higher predictive accuracy and robustness of XGBoost compared to Random Forest.

In contrast, Random Forest uses the Bagging (Bootstrap Aggregating) algorithm [24], which creates multiple decision trees that are trained independently on random subsets of the data. The final prediction is then made by averaging or taking a majority vote from these trees. While this approach is computationally less intensive and can be parallelized, it generally offers fewer hyperparameters to fine-tune, potentially limiting its optimization capacity. Random Forest also randomly selects a subset of features for each tree, adding a layer of robustness but possibly missing out on the nuanced feature selection that XGBoost offers through its gradient information.

Furthermore, XGBoost has a more extensive range of hyperparameter options, providing greater flexibility to adapt to various data types and problem complexities [24]. This is especially beneficial in scenarios requiring intricate model tuning. Both algorithms consider the importance of features in making predictions, but XGBoost refines this process by optimizing based on prediction errors and incorporating regularization terms in its information gain calculations, leading to more accurate and insightful feature selection.

In summary, while both XGBoost and Random Forest are powerful ensemble learning techniques, the choice between them often depends on the specific requirements of the project, including the nature of the data, computational resources, and the level of interpretability needed. XGBoost generally excels in scenarios requiring high predictive accuracy and intricate model tuning, whereas Random Forest may be more suitable for projects that prioritize interpretability and computational efficiency. Therefore, the effectiveness of each algorithm is highly context-dependent and should be considered carefully based on the project’s unique needs.

While the focus of this study has been on the predictive accuracy of Random Forest and XGBoost models, it’s important to also consider the computational cost and time efficiency of these algorithms, especially when deployed in real-world clinical settings. Random Forest, with its parallelizable nature, can be more time-efficient and less computationally demanding, making it a viable option for systems with limited computational resources. On the other hand, the sequential nature of XGBoost’s Gradient Boosting can be computationally intensive and time-consuming, particularly for large datasets or complex feature spaces. Therefore, the choice between these two algorithms may also hinge on the available computational resources and the urgency of obtaining predictive results. This aspect could be particularly crucial in medical applications where timely decision-making is often required but was not explored in the current study.

We acknowledge a critical limitation regarding the sample size and validation methods of our study. It involved a relatively small cohort of 57 patients, all treated at Kaohsiung Chang Gung Memorial Hospital. This limited sample size might affect the generalizability of our findings across different populations and settings. Although our models underwent rigorous internal validation using 10 iterations of 10-fold cross-validation to ensure reliability and robustness within our dataset, the lack of external validation could restrict confirmation of these models’ robustness and broader applicability.

Regarding the minimal differences in AUC scores among the logistic regression (AUC = 0.870), XGB_ALL_ (AUC = 0.890), and XGB_LASSO_ (AUC = 0.820) models, we acknowledge the constraints posed by our small sample size and single-institution study design. While the incremental gains in predictive performance with ensemble methods are evident, their clinical significance remains to be fully substantiated.

The advantages of ensemble methods, as reflected in the slightly higher AUC for XGB_ALL_, suggest potential improvements in handling complex interactions between clinical and dosimetric variables, which are not as effectively captured by logistic regression. However, the modest enhancements observed warrant a cautious interpretation regarding the generalizability and practical application of these findings.

Recognizing the limitations of our study, it is crucial to conduct further research involving a larger, more diverse patient cohort across multiple institutions. External validation of our models is essential to confirm their robustness and assess their performance in broader clinical contexts. Such studies would not only provide a definitive evaluation of the models’ utility in clinical practice but also help establish more reliable benchmarks for predicting radiation dermatitis. This would strengthen the evidence for using advanced machine learning techniques in clinical settings and potentially lead to more personalized and effective management strategies for patients undergoing proton radiotherapy.

To enhance the credibility and applicability of our predictive models, future research should aim to include a more diverse patient cohort from multiple institutions. This expansion would provide a robust evidence base and facilitate essential external validation, verifying the models’ effectiveness across various clinical environments. This step is crucial for ensuring that our predictive models can reliably support clinical decision-making and improve patient outcomes in diverse settings. Furthermore, we recommend fostering collaboration between centers to enrich the datasets, allowing for nuanced adjustments and calibrations of the models according to different patient demographics and treatment protocols. Such collaboration would enhance the predictive accuracy and clinical utility of our risk assessment tools, making them more effective in practical applications.

## Conclusion

This study evaluates the efficacy of traditional and ensemble machine learning methods for predicting radiation dermatitis in head and neck cancer patients after proton radiotherapy. Ensemble machine learning, particularly XGBoost with its inherent feature selection capabilities, outperforms logistic regression, which requires complex procedures like LASSO for preventing overfitting. XGBoost simplifies model construction by integrating feature convergence within its algorithm, eliminating the need for additional steps like LASSO, thus streamlining the modeling process while achieving comparable or superior performance.

Despite these advantages, it’s crucial to note that the skin data was collected retrospectively from patient charts, which might limit data quality. While acute radiation dermatitis significantly impacts patients, it is not the primary factor guiding treatment decisions in this cohort. Therefore, the practical utility of these predictive models in clinical settings requires careful validation to ensure they truly support improved patient management and decision-making. This study highlights that while logistic regression is useful for exploratory analysis due to its simplicity, ensemble methods offer a more efficient and potentially more accurate approach for clinical applications, pending further validation.

## Data Availability

The datasets used and/or analyzed during the current study available from the corresponding author on reasonable request.

## References

[1] Hegedus F, Mathew LM, Schwartz RA (2017). Radiation dermatitis: an overview. Int J Dermatol.

[2] Kang J, Schwartz R, Flickinger J, Beriwal S (2015). Machine learning approaches for predicting radiation therapy outcomes: a clinician’s perspective. Int J Radiation Oncology* Biology* Phys.

[3] Gao S, Calhoun VD, Sui J (2018). Machine learning in major depression: from classification to treatment outcome prediction. CNS Neurosci Ther.

[4] Samant P, de Ruysscher D, Hoebers F, Canters R, Hall E, Nutting C, Maughan T, Van den Heuvel F. Machine learning for normal tissue complication probability prediction: predictive power with versatility and easy implementation. Clin Translational Radiation Oncol 2023, 39. 10.1016/j.ctro.2023.100595 PMC9984444 36880063

[5] Lee T-F, Liou M-H, Huang Y-J, Chao P-J, Ting H-M, Lee H-Y, Fang F-M (2014). LASSO NTCP predictors for the incidence of xerostomia in patients with head and neck squamous cell carcinoma and nasopharyngeal carcinoma. Sci Rep.

[6] Babyak MA (2004). What you see may not be what you get: a brief, nontechnical introduction to overfitting in regression-type models. Psychosom Med.

[7] Dietterich TG. Ensemble methods in machine learning. In: *International workshop on multiple classifier systems: 2000*: Springer; 2000: 1–15.

[8] Zhang C, Ma Y. Ensemble machine learning: methods and applications. Springer; 2012.

[9] Liao K-C, Huang Y-J, Tsai W-L, Lee C-H, Fang F-M (2024). Longitudinal assessment of quality of life in nasopharyngeal cancer patients treated with intensity-modulated proton therapy and volumetric modulated arc therapy at different time points. Cancers.

[10] Yokota T, Zenda S, Ota I, Yamazaki T, Yamaguchi T, Ogawa T, Tachibana H, Toshiyasu T, Homma A, Miyaji T (2021). Phase 3 randomized trial of topical steroid versus placebo for prevention of radiation dermatitis in patients with head and neck cancer receiving chemoradiation. Int J Radiation Oncology* Biology* Phys.

[11] Mangalathu S, Hwang S-H, Jeon J-S (2020). Failure mode and effects analysis of RC members based on machine-learning-based SHapley Additive exPlanations (SHAP) approach. Eng Struct.

[12] Bertsimas D, Gupta V, Kallus N (2018). Data-driven robust optimization. Math Program.

[13] Lee TF, Chao PJ, Ting HM, Chang LY, Huang YJ, Wu JM, Wang HY, Horng MF, Chang CM, Lan JH et al. Using Multivariate Regression Model with least Absolute Shrinkage and Selection Operator (LASSO) to predict the incidence of Xerostomia after Intensity-Modulated Radiotherapy for Head and Neck Cancer. PLoS ONE 2014, 9(2). 10.1371/journal.pone.0089700 PMC3938504 24586971

[14] Kong C, Zhu X-z, Lee T-F, Feng P-b, Xu J-h, Qian P-d, Zhang L-f, He X, Huang S-f (2016). Zhang Y-q: LASSO-based NTCP model for radiation-induced temporal lobe injury developing after intensity-modulated radiotherapy of nasopharyngeal carcinoma. Sci Rep.

[15] Satheeshkumar PS, El-Dallal M, Mohan MP (2021). Feature selection and predicting chemotherapy-induced ulcerative mucositis using machine learning methods. Int J Med Informatics.

[16] Smith DK, Clark H, Hovan A, Wu J (2023). Neural network and spline-based regression for the prediction of salivary hypofunction in patients undergoing radiation therapy. Radiat Oncol.

[17] Xie Y, Wang Q, Hu T, Chen R, Wang J, Chang H, Cheng J (2021). Risk factors related to acute radiation dermatitis in breast cancer patients after radiotherapy: a systematic review and meta-analysis. Front Oncol.

[18] Lilla C, Ambrosone CB, Kropp S, Helmbold I, Schmezer P, von Fournier D, Haase W, Sautter-Bihl M-L, Wenz F, Chang-Claude J (2007). Predictive factors for late normal tissue complications following radiotherapy for breast cancer. Breast Cancer Res Treat.

[19] Fang KC, Lee CH, Chuang HC, Huang TL, Chien CY, Tsai WL, Fang FM (2023). Acute radiation dermatitis among patients with nasopharyngeal carcinoma treated with proton beam therapy: prognostic factors and treatment outcomes. Int Wound J.

[20] Sagi O, Rokach L (2018). Ensemble learning: a survey. Wiley Interdisciplinary Reviews: Data Min Knowl Discovery.

[21] Maalouf M (2011). Logistic regression in data analysis: an overview. Int J Data Anal Techniques Strategies.

[22] Mao S, Chen J-W, Jiao L, Gou S, Wang R (2019). Maximizing diversity by transformed ensemble learning. Appl Soft Comput.

[23] Chen T, Guestrin C. Xgboost: A scalable tree boosting system. In: *Proceedings of the 22nd acm sigkdd international conference on knowledge discovery and data mining*: 2016; 2016: 785–794.

[24] Syam N, Kaul R. Random forest, bagging, and boosting of decision trees. machine learning and artificial intelligence in Marketing and sales: essential reference for practitioners and data scientists. edn.: Emerald Publishing Limited; 2021. pp. 139–82.

